# Editorial: Identifying genetics-based mechanisms and treatments for neurodevelopmental and psychiatric disorders through data integration

**DOI:** 10.3389/fgene.2023.1186489

**Published:** 2023-04-03

**Authors:** Kaifang Pang, Li Wang, Suhua Chang

**Affiliations:** ^1^ Department of Pediatrics, Baylor College of Medicine, Houston, TX, United States; ^2^ Jan and Dan Duncan Neurological Research Institute, Texas Children’s Hospital, Houston, TX, United States; ^3^ The Eli and Edythe Broad Center of Regeneration Medicine and Stem Cell Research, University of California, San Francisco, San Francisco, CA, United States; ^4^ Department of Neurology, University of California, San Francisco, San Francisco, CA, United States; ^5^ Peking University Sixth Hospital, Peking University Institute of Mental Health, NHC Key Laboratory of Mental Health (Peking University), National Clinical Research Center for Mental Disorders (Peking University Sixth Hospital), Beijing, China; ^6^ Chinese Academy of Medical Sciences Research Unit (No 2018RU006), Peking University, Beijing, China

**Keywords:** disease genetics, disease mechanism, single cell, network, drug

Recent genome-wide association and exome/genome sequencing studies have revolutionized our understanding of the genetic architecture of complex diseases including neurodevelopmental and psychiatric disorders. However, the biological mechanisms underlying disease pathology remain largely unknown, hindering the development of effective therapies. On the other hand, recent technological advances have made diverse functional data increasingly available. The functional data include spatiotemporal transcriptome of the brain, single-cell RNA sequencing (scRNA-seq) data of the brain, transcription factor-target gene network, protein-protein interaction network, functional interaction network, Gene Ontology, biological pathways, drug-target gene network, drug-target gene expression data, and so on. These rich functional data, especially scRNA-seq data, offer us a great resource to dissect disease genetics, elucidate molecular mechanisms, and discover treatment strategies through data integration.

Several computational studies have already made progress in this direction by integrating disease genetics with scRNA-seq data of the brain. For example, Skene et al. performed cell-type specificity analyses by integrating genome-wide association analysis results of schizophrenia with scRNA-seq data of the brain. They found that schizophrenia genes are specifically expressed in certain brain cell types (Skene et al., 2018). Pang et al. developed a coexpression enrichment analysis approach by integrating risk genes involved in several neurodevelopmental disorders with scRNA-seq data of the developing human prefrontal cortex. They identified key brain cell types and cellular processes disrupted in specific neurodevelopmental disorders (Pang et al., 2020). Mohammadi et al. developed a computational framework to reconstruct cell-type-specific interactomes based on scRNA-seq data of the adult human frontal cortex and a reference interactome. They discovered that risk genes in specific brain disorders tend to interact in specific brain cell types (Mohammadi et al., 2019). Aibar et al. developed a computational method to reconstruct gene regulatory networks based on scRNA-seq data of the brain and regulatory sequence data (Aibar et al., 2017). Further integration of the inferred gene regulatory networks with disease genetics could provide new insights into disease mechanisms.

This Research Topic aims to collect articles related to data integration to identify genetics-based mechanisms and treatments for developmental, neurological, and psychiatric disorders. Five high-quality articles have been selected in this collection, and we believe that these five articles have valuable contributions to this direction.

Drozd et al. performed whole exome sequencing analysis of nine trios with early-onset schizophrenia and comorbid neurodevelopmental conditions. They prioritized variants and genes using multiple bioinformatic tools and found that the candidate genes tend to interact in a protein-protein interaction network and fall within the Wnt, cadherin, and cholecystokinin receptor signaling pathways. This unique study identified the genetic component underlying a specific group of schizophrenia (early-onset schizophrenia), which helps us better understand the genetic basis of diverse types of schizophrenia.

Guan et al. proposed integrative regularized non-negative matrix factorization by imposing a new regularization based on integrative non-negative matrix factorization. They then used the method to identify shared and cell-type-specific gene modules from gene expression data of multiple human brain cell types. They further performed module enrichment analysis of risk genes in autism spectrum disorder (ASD) and identified the shared and cell-type-specific gene modules enriched with ASD genes. They found that the shared ASD-associated gene modules are enriched in synaptic function, whereas the cell-type-specific ASD-associated gene modules tend to have distinct functions. This study uncovered the shared and cell-type-specific functions of ASD genes, which has important contributions to our understanding of ASD pathophysiology at the single-cell level.

Cerebellar dysfunction is thought to contribute to the sensory and social impairments in ASD (Tsai et al., 2012). Brandenburg et al. aimed to understand the transcriptional changes in the cerebellar Purkinje cells in ASD patients. To this end, they performed laser capture microdissection to isolate pure populations of cerebellar Purkinje cells in postmortem ASD brain samples and control samples. They then performed RNA sequencing and found that genes related to connectivity, extracellular matrix, calcium signaling, and immune function are altered in the Purkinje cells of ASD patients. This sort of cell-type-specific bulk-level transcriptomic profile provides a higher sensitivity relative to scRNA-seq data and allows the enrichment of rare populations for detailed analysis.

Data integration and network analysis for neurodevelopmental disorders are often done at the gene level. However, it is known that different pathogenic variants in the same gene could lead to diseases with various manifestations. Thus, understanding the mutational spectrum of known disease genes and their correlation to disease phenotypes is crucial for identifying pathogenic mechanisms (Wang et al., 2019). Liu et al. performed a retrospective analysis on 80 subjects with retinal dystrophy and rare variants in *RPGR*, a gene frequently mutated in X-linked retinitis pigmentosa. Their results identified six reported and 28 novel variants in this gene. Among them, ∼70% were classified as likely pathogenic. The study expands the known mutational spectrum of *RGPR*, which contributes to a better understanding of pathogenic mechanisms and genetic diagnosis.

Besides protein-coding genes, non-coding RNAs may also join the regulation network, contributing to the functions and dysfunctions of genes as well as the pathogenic mechanisms of diseases. Furthermore, mRNAs and non-coding RNAs could be used as biomarkers for disease prediction. Zhang et al. used a competing endogenous RNAs (ceRNA) expression dataset and an mRNA expression dataset in trabecular meshwork from patients with Primary Open-Angle Glaucoma to identify differentially expressed ceRNAs and mRNAs. They further constructed a ceRNA-miRNA-mRNA network and identified five core genes. The core genes, which were associated with immune infiltration, not only can help understand the disease mechanisms but also have the potential to be used as diagnostic biomarkers for disease prediction. These data integration analyses could provide clues for disease mechanisms and diagnoses.

While genetic studies continue to increase our understanding of the genetic basis of complex diseases and diverse functional data are growing unprecedentedly fast, we think now is the best time to develop computational methods for data integration to translate disease genetics into mechanisms to further guided treatments. We hope that this collection of articles can inspire more researchers to work on this direction, and we believe that our joint efforts will lead to novel therapies for many complex genetic diseases in the near future.
